# Distractions in digital reading: a meta-analysis of attentional interference effects

**DOI:** 10.3389/fpsyg.2025.1671214

**Published:** 2025-11-19

**Authors:** Yamin Shen

**Affiliations:** Department of Chinese Language and Literature, East China Normal University, Shanghai, China

**Keywords:** attention interference, online environment, reading comprehension, moderating effect, meta-analysis

## Abstract

**Introduction:**

Digital reading has become a common learning activity; however, the empirical understanding of how attentional interference affects comprehension remains limited.

**Methods:**

This study employed a meta-analytic approach to synthesize 32 empirical studies investigating the effects of attentional distractors in digital environments. The analysis focused on the overall comprehension effects of attentional distraction in online reading contexts, while examining potential moderating variables including study design, type of attentional distraction, required effort, time control, type of article, participant age, reading device, and time of publication.

**Results:**

The research results indicate that Hedges’ g is -0.6411, suggesting a negative effect of attentional interference in networked environments on reading comprehension. Moderation analysis further shows that factors such as research design, type of distraction, and educational level influence this effect, particularly in between-subject studies involving television (TV) or music distractions, where older readers experienced greater declines in comprehension.

**Conclusion:**

This study advances the understanding of the cognitive-neurological foundations of attention and cognitive load theory, emphasizing the limited capacity of attention in online environments and the need to balance cognitive load to improve reading comprehension. It encourages minimizing distractions such as background music and videos, designing interactive online platforms that promote focus and selfregulated learning, using tools such as eye-tracking to monitor attention, and implementing tailored interventions that help students develop effective selfregulation strategies for better comprehension.

## 1 Introduction

With the widespread adoption of the Internet, the use of media for various tasks in networked environments has become increasingly common (Luo et al., 2020). The digital reading environment comprises a virtual space supported by devices such as computers, tablets, smartphones, and televisions (TVs), and the advantages of these digital media provide opportunities for interactivity and versatility (Schwabe et al., 2022). Attentional interference refers to the diversion of attention from a primary task to other tasks, whether related or unrelated (Wang, 2022). In the context of reading, assessments and their items can generally be categorized into those that focus on word comprehension and those that assess reasoning, both of which are commonly evaluated using quizzes in relevant studies (Chung et al., 2017; Mardini et al., 2024).

### 1.1 Impact of attentional interference on reading comprehension in digital and multitasking environments

According to existing research, attentional interference in online environments has produced mixed effects on reading comprehension, with some studies reporting predominantly negative effects, others reporting positive effects, and some showing no significant impact.

Various patterns of attentional interference generally impair reading comprehension, often leading to lower comprehension task performance. Specifically, different types of auditory distraction—such as external sounds encountered during reading—can influence comprehension outcomes. According to the attention and cognitive load theory, humans possess limited cognitive resources that must be efficiently allocated across tasks (Lang, 2017). When individuals are exposed to competing stimuli or simultaneous tasks, attentional resource distribution becomes strained, resulting in reduced processing efficiency. For instance, while reading, the use of smartphones and exposure to smartphone notifications can distract students and reduce their reading comprehension scores (O'Toole, 2024). According to research, attentional interference that requires conscious effort can negatively affect reading comprehension in online environments compared with passive forms of distraction, such as simple pop-up windows (Luke and Jensen, 2023). In digital settings, visual distractions, such as advertisements, reduce reading speed and impair text processing efficiency (Lan et al., 2025). In networked environments, multitaskers often experience frequent media-related interruptions, which can disrupt the reading process and diminish comprehension effectiveness (Chevet et al., 2022b). Moreover, when reading online, internal distractions, such as mind-wandering, can also impair comprehension (Bonifacci et al., 2023). Research further indicates that reading comprehension becomes less effective as the cognitive load required by distractions increases (Velázquez Castillo, 2024).

Previous studies have shown that certain forms of attentional interference can positively influence reading comprehension, as evidenced by improved standardized comprehension assessment scores. Conversely, Sweller (1994) emphasized that processing multiple tasks within the same timeframe increases cognitive load, thereby diminishing task performance. Additionally, the working memory model recommends that individuals with higher working memory capacity are better equipped to filter out irrelevant distractions and maintain focus, whereas those with lower capacity are more susceptible to attentional interference (Hughes, 2014). For instance, Hao and Conway (2022) systematically manipulated cognitive load by varying background noise conditions and investigated individual differences in working memory capacity. Their findings indicated that participants with high perceptual load and strong working memory resources demonstrated greater accuracy in reading comprehension tasks, underscoring the interactive effects of cognitive load and memory capacity on comprehension outcomes. Multimedia and interactive features in online environments can enhance reading comprehension (Schwabe et al., 2022). In networked settings, interference may also have a facilitating effect when participants possess relevant prior knowledge, which supports text comprehension (Ronconi et al., 2025).

However, other studies have found that certain types of attentional interference have no significant effect on reading comprehension, as indicated by unchanged comprehension scores. For example, Chevet et al. (2022a) found that interruptions during reading did not impair the comprehension of procedural texts, as participants demonstrated similar performance when test information was matched against the content presented at the point of interruption. Additionally, Ronconi et al. (2025) reported that simple static screen distractions had minimal impact on the reading comprehension of college students during digital reading tasks.

Overall, empirical evidence on the effects of attentional interference on online reading comprehension remains inconsistent. This study quantitatively examines the overall impact of attentional interference on reading comprehension in online settings from 2000 to 2025. Additionally, it identifies key moderating variables and seeks to confirm that certain types of interference, such as music and TV, serve as core moderators. These findings indicate that different forms of interference are processed differently by the brain’s cognitive mechanisms, offering experimental support for cognitive load theory as it relates to attentional interference. This study also incorporates real-world elements, such as social media interactions, to deepen the understanding of digital reading behavior by focusing on reading comprehension within authentic online environments.

### 1.2 Potential moderating variables influencing the impact of attentional interference on reading comprehension

**Figure 1:**
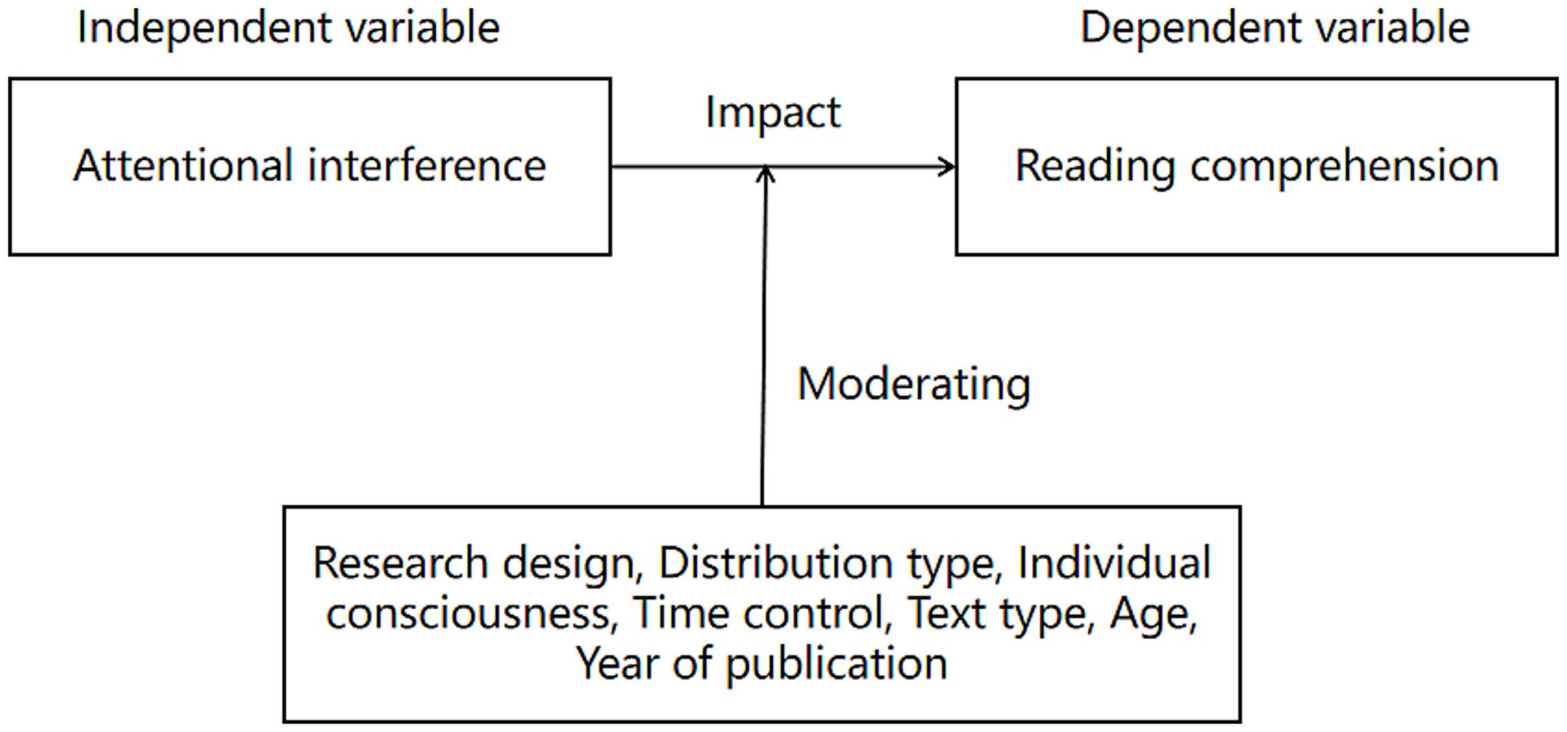
Research framework.

The following moderating variables are considered in this meta-analysis, as illustrated in Figure 1. The study design is categorized as either between-group or within-group. Types of attentional interference include instant messaging (IM), reading other materials, watching TV, and listening to background music. Individual consciousness is categorized based on whether conscious effort is required to complete the task or not. Time control indicates whether a time limit is required to complete the primary tasks. The text type is categorized as narrative or expository. The participants were classified into age-based groups corresponding to elementary school, middle school, high school, college, and adult learners. Reading devices are categorized as either paper- or computer-based. The publication date of the journal is also included as a moderating variable. These variables have been incorporated into this study based on a prior study indicating that they may moderate the effects of attentional interference on reading comprehension in digital contexts. Further explanation is provided below.

#### 1.2.1 Research design

Tran et al. (2025) observed that the reading comprehension outcomes varied across three variations of within-group experimental designs. To account for methodological variability and ensure comparability across studies, this meta-analysis distinguishes between two primary research designs: between-group and within-group. In the between-group design, participants were assigned to two groups to compare the effects of attentional interference and non-interference on reading comprehension in an online environment. In contrast, the within-group design exposed the same participants to both attentional interference and non-interference conditions, enabling direct comparison of comprehension performance across differing attentional conditions.

#### 1.2.2 Type of interference

Attentional interference can adversely affect reading comprehension (Velázquez Castillo, 2024). However, background noise and IM do not always disrupt reading performance (Fante et al., 2013). Therefore, this study hypothesized that the effects of attentional interference on reading comprehension differ based on the interference type, categorized into five experimental conditions: IM, reading other materials, watching TV, background music, and environmental noise. Distractions encountered in online environments can influence the neural processes underlying reading comprehension. Different types of interference are associated with varying cognitive load levels and elicit distinct patterns of brain activity related to semantic processing (Ye et al., 2022; Veerasingam et al., 2024). For instance, background music primarily imposes auditory stimulation; TV induces multimodal (visual and auditory) interference; and information-based distractions, such as IM, result in internal competition for attentional resources. Factors such as individual differences, task characteristics, and cognitive load jointly moderate how each interference type influences reading comprehension.

#### 1.2.3 Individual consciousness

Reading comprehension in online environments is often disrupted by competing tasks, particularly when those tasks consciously demand significant mental effort and attention. Activities that require deliberate cognitive engagement—such as listening to music—have been shown to impair reading comprehension more significantly than multitasking behaviors that do not demand sustained attention (Jeong and Hwang, 2012). Accordingly, assessing whether the secondary task also requires active mental effort is important. On this basis, a moderating effect was hypothesized in the relationship between attentional interference and reading comprehension, depending on whether the task required higher allocation of cognitive resources and focused attention.

#### 1.2.4 Time control

Empirical evidence shows that time pressure increases cognitive load, thereby hindering task performance (Barrouillet et al., 2007). However, some studies have proposed that time pressure may induce a beneficial form of cognitive load—referred to as “desirable” cognitive load—by enhancing deeper task engagement. Therefore, it was anticipated that reading comprehension would be more effective under conditions without time constraints and that the effects of attentional interference would be further amplified when time pressure was present in digital reading environments.

#### 1.2.5 Text type

Different texts can be classified into two categories based on textual characteristics: illustrative and narrative (Graesser and McNamara, 2011). Previous research has shown that readers demonstrate higher comprehension when engaging with narrative texts compared to illustrative ones (Mar et al., 2021). Conversely, other findings indicate that individuals reading on digital screens often exhibit better comprehension with illustrative than narrative text (Mao et al., 2023). Given these contrasts, screen-based reading was expected to yield superior comprehension for illustrative texts relative to narrative texts, and this disparity would be more evident under conditions of attentional interference in online environments.

#### 1.2.6 Age

Prior research demonstrated a negative correlation between the frequency of digital media usage and reading comprehension performance among adolescents, indicating that increased engagement with digital platforms is associated with diminished comprehension abilities (Duncan et al., 2016). Empirical evidence further indicates that while multimedia elements foster reading comprehension in elementary-aged children, the presence of interactive features can impair attentional focus during reading tasks (Takacs et al., 2015). To examine the influence of attentional interference on digital reading comprehension, participants were stratified into four educational cohorts based on age: elementary, secondary, university, and adult learners. Attentional interference was hypothesized to exert a more substantial detrimental effect on reading comprehension in older adults than in younger age groups.

#### 1.2.7 Reading device

Chen and Catrambone (2015) reported that students demonstrated no significant differences in reading comprehension outcomes between print- and screen-based reading modalities. However, a meta-analysis conducted by Kong et al. (2018) concluded that reading from printed texts is more resistant to attentional distractions than reading from digital screens. Accordingly, this study examines the impact of attentional interference levels on reading comprehension performance across both digital and print formats, with particular attention to the interference of the online environment, an external variable that warrants further investigation.

### 1.3 Research problem

Previous empirical studies have identified three primary patterns of attentional interference effects on reading comprehension in digital contexts: significant positive, significant negative, and non-significant effects. The principal research question guiding this study was as follows: What is the cumulative effect of attentional interference on reading comprehension in online environments? Drawing on the findings from the aforementioned literature, multiple moderating variables often influence the relationship between distraction and reading comprehension in online environments. Subsequently, the secondary research question posed was: Which factors moderate the effect of attentional interference on reading comprehension in online environments?

## 2 Research methodology

This study adopted a meta-analytic approach to synthesize existing empirical findings and evaluate the influence of attentional distractions on reading comprehension in online contexts. This method enhances the precision and reliability of the conclusions, thereby increasing their scientific validity and broader applicability. The meta-analysis followed five systematic phases: (1) defining the research objectives; (2) conducting a comprehensive literature screening based on predefined inclusion and exclusion criteria to identify pertinent studies; (3) defining moderating variables and coding the selected literature; (4) extracting and inputting relevant data from the study; and (5) conducting statistical analyses and reporting the findings.

### 2.1 Search for relevant studies

A comprehensive literature search across Chinese and foreign language databases was conducted to identify relevant studies published between January 2000 and February 2025. The search was initiated in 2000, as social media technologies had not yet been widely integrated into everyday reading practices. Restricting the search to publications from 2000 onward enables a focused examination of the evolving impact of digital distraction on reading comprehension over time (Ronconi et al., 2025). The search period concluded in February 2025 to ensure the inclusion of the most recent research and capture recent trends in the influence of distraction on reading comprehension in online environments (Kimura and Nakashima, 2025).

An initial search was conducted across five major English academic databases: Web of Science, EBSCO, SpringerLink, Scopus, and ProQuest. Supplementary searches were subsequently performed using Google Scholar to enhance the comprehensiveness of the retrieval process. Additionally, the China National Knowledge Infrastructure database was queried to incorporate relevant Chinese literature. The following keywords were employed in the preliminary search: network environment, watch out for interference, and reading comprehension. Boolean operators, parentheses, and wildcards were then used to construct complex query strings, including multitasking AND reading AND comprehension, multitasking AND comprehension, digital device AND comprehension, multitasking AND reading, multitasking AND reading, task switch AND reading, distraction AND reading, distraction AND comprehension. These queries were applied to the title, abstract, and keywords to maximize relevant literature retrieval. All English language query formulations were appropriately translated and adapted for the Chinese database to ensure consistency and inclusivity in the search strategy.

### 2.2 Inclusion criteria for the selected studies

The meta-analysis in this study followed the methodological frameworks established by Glass et al. (1981), Lipsey and Wilson (2001), and Borenstein et al. (2009). Meta-analysis integrates findings from studies of varying methodological rigor to produce a comprehensive, evidence-based conclusion. The following inclusion and exclusion criteria were established to identify suitable studies for this meta-analysis: (1) The study must focus on the effect of distraction on reading comprehension in an online environment. As such, it should indicate whether participants engaged in online reading, describe the distraction strategies employed, and report reading comprehension scores for both the control and experimental groups. (2) To ensure that differences in comprehension outcomes are attributable to the distraction strategy alone, participants must have been randomly assigned to control and experimental groups, with efforts made to control for confounding variables such as age and educational backgrounds. Studies without random assignment were excluded. (3) The study must report key methodological variables, including study design, time constraints, and article type. (4) Reading comprehension data must be quantitative and include sufficient statistical information (e.g., means, standard deviations, or standard errors) for both control and experimental groups to calculate the effect size. Studies that only presented comparative outcomes between groups without providing the underlying statistical data were excluded.

**Figure 2:**
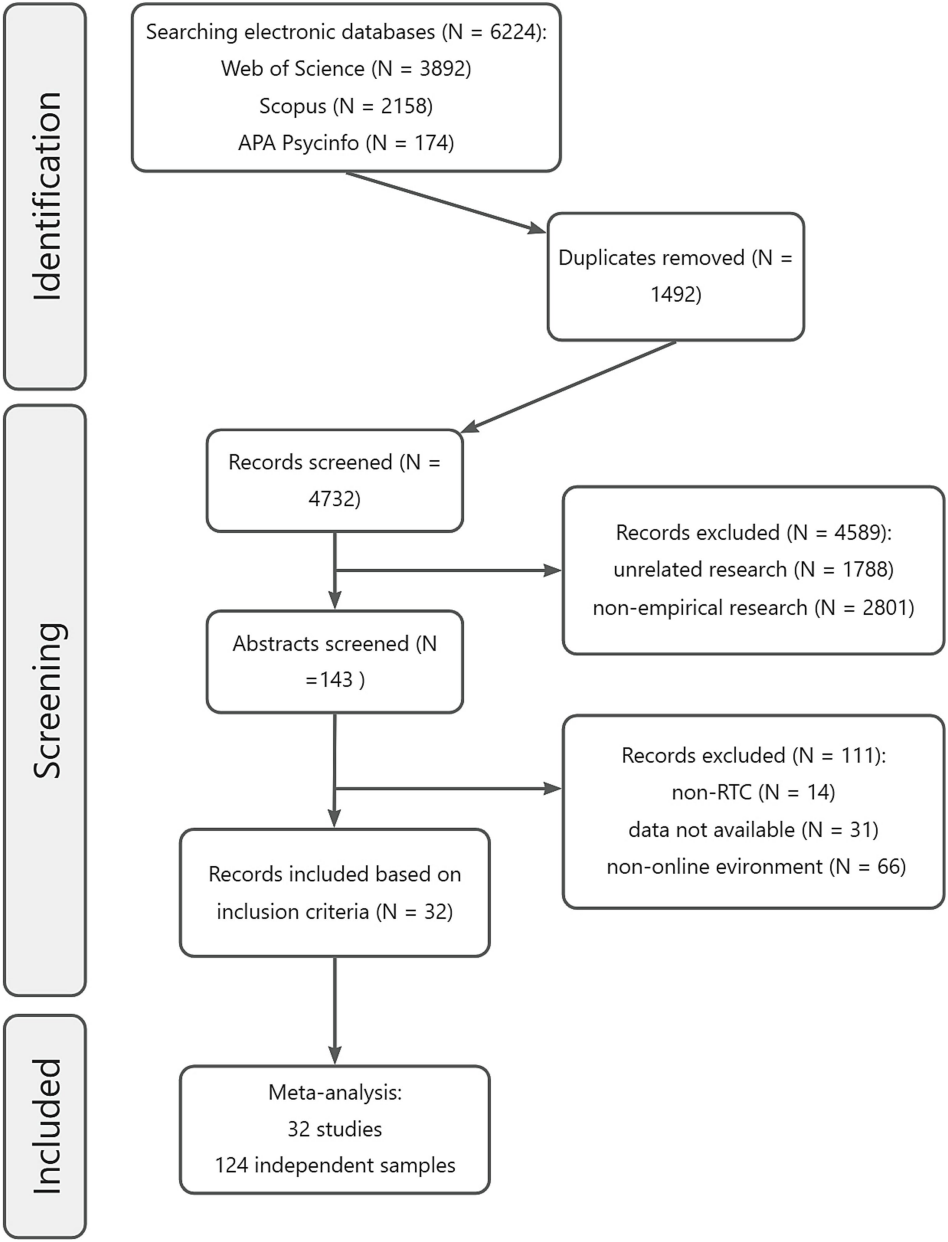
Flow diagram of the literature search process.

An advanced search strategy was employed to collect literature from mainstream academic databases, restricting the language to English and publication dates to between January 1, 2000, and January 1, 2025. The search was conducted across three databases: (1) Scopus, using the following search expression: (TITLE-ABS-KEY (multitask AND read AND comprehension) OR (multitasking AND comprehension) OR (digital AND device AND comprehension) OR (task AND switch AND reading) OR (distraction AND (reading OR comprehension))), which retrieved 2,158 articles; (2) Web of Science, using a similar search expression, retrieved 3,892 articles; and (3) APA PsycInfo, which retrieved 174 articles using a comparable query. In total, 6,224 articles were initially identified. After removing duplicates using EndNote, 4,732 articles were screened. Figure 2 shows the complete screening and selection process.

### 2.3 Coding of the studies

A standardized coding protocol was applied to all included studies to determine whether sufficient variables and metrics were provided for subsequent subgroup analyses. Only studies with adequate data were eligible for subgroup comparisons. For a study to be included in the subgroup analysis, it had to report information on the following characteristics: (1) time of publication, (2) study design, (3) type of attentional distraction, (4) required effort, (5) time control, (6) type of article, (7) participant age, and (8) reading device. Table 1 presents the specific values for the following variables:

**Table 1:** Coding criteria.

Number	Typology	Encodings
1	Time of manuscript publication	From 2000 to 2025
2	Study design	Between-group and within-group studies
3	Type of attention distraction	Messaging, reading other material, watching TV, listening to background music, and background noise
4	Effort consciousness	Need, no-need
5	Time control	Limit, no-limit
6	Type of article	Narrative and expository writing
7	Age	Elementary school, middle school, high school, college, and adult learners
8	Reading device	Paper, computer
9	Publish year	Year of publication of the literature
10	Sample size of the control group	Number of participants who were not exposed to attentional interference in the sample set
11	Mean reading comprehension score of the control group	Mean reading comprehension score for participants not exposed to attentional interference
12	Standard deviation of reading comprehension scores in the control group	Standard deviation of reading comprehension scores for participants not exposed to attentional interference
13	Sample size and mean reading comprehension score of the experimental group	Number of participants who were exposed to attentional interference in the sample set
14	Mean reading comprehension score in the experimental group	Mean reading comprehension score for participants exposed to attentional interference
15	Standard deviation of reading comprehension scores in the experimental group	Standard deviation of reading comprehension scores for participants exposed to attentional interference

Codes for Types 2–9 were used as moderating variables in the subgroup analyses.

### 2.4 Estimation of the effect sizes

In line with standard practices in meta-analytic research, this study defined the effect size as the Standardized Mean Difference (SMD), which standardizes mean differences between the intervention and control groups using the pooled within-group standard deviation. The SMD is scale-independent, allowing for comparisons across studies regardless of the instruments used to assess reading comprehension. Hedges’ g was employed to estimate the effect size because of its widely accepted correction for small sample bias. All included studies provided the necessary data, means, standard deviations, SEs, and sample sizes for both the intervention and control groups to meet the requirements for calculating the effect size. This ensured that effect sizes could be systematically computed across all studies included in the meta-analysis. Subgroup analyses were also conducted to explore the potential moderating effects. This study employed a mixed-effects model for analysis. A random-effects model was used to aggregate studies within each subgroup based on the assumption of true effect size heterogeneity among studies within the same subgroup. Conversely, a fixed-effects model was applied to aggregate effect sizes across subgroups because the subgroup levels were predetermined and not randomly selected. In this context, “fixed” refers to the deliberate selection of subgroup categories, allowing for comparison across clearly defined, non-random groups.

## 3 Findings

A total of 124 experiments from 32 studies with publication dates ranging from 2000 to 2025 were included in the meta-analysis. Regarding study design, 89 experiments employed a between-group design, whereas 35 used a pretest-posttest design within groups. In terms of attentional interference types in the online environment, five distinct categories were identified across the 124 experiments: 13 experiments used IM, 59 used watching TV, 25 involved listening to music, 19 used interruption-based distractions, and 8 used background noise. These classifications allowed for detailed subgroup analysis of the effect of distraction types on reading comprehension outcomes.

At the college level, 40 experiments involved participants using computers as reading devices, whereas 84 involved participants reading from paper. Regarding attentional demands, participants in 89 experiments were required to exercise conscious effort to manage the distractor, whereas those in 35 experiments experienced passive distraction without the need for conscious effort. Regarding time constraints, 82 experiments imposed time limits on reading comprehension tasks, whereas 42 allowed participants to complete the tasks without time limits. For reading comprehension assessment, 38 experiments assigned participants narrative texts, and 86 were assigned expository texts, allowing for comprehension performance evaluation across different text types. Three experiments involved elementary school students, four involved middle school students, and 11 involved high school students. Lastly, 106 of the experiments used paper as the reading medium, indicating a strong representation of traditional reading formats across age groups.

### 3.1 Heterogeneity test

Inter-study heterogeneity in the meta-analytic dataset was evaluated using data from 124 experiments across 32 included studies using Cochran’s *Q*-test and the *I*^2^ statistic. A high *Q*-statistic, along with a low *p*-value, indicates heterogeneity and significant variability in effect sizes beyond what would be expected by chance. The *I*^2^ statistic quantifies the percentage of total variance in effect sizes due to true differences between studies rather than random error. As shown in Table 2, the *Cochran’s Q-test* yielded a statistically significant result (*p* < 0.001), and the *I*^2^ value indicated that 88.3% of the variability was attributable to inter-study differences. These results provide strong evidence of substantial heterogeneity among the included studies.

**Table 2:** Results of the heterogeneity analysis.

*I* ^2^	*H*	*Q*	*p*
88.3%	2.93	1054.43	<0.001

*I*^2^ statistic tells how much the effect size varies across studies. The high *I*^2^ index measures considerable heterogeneity.

### 3.2 Publication bias test

**Figure 3:**
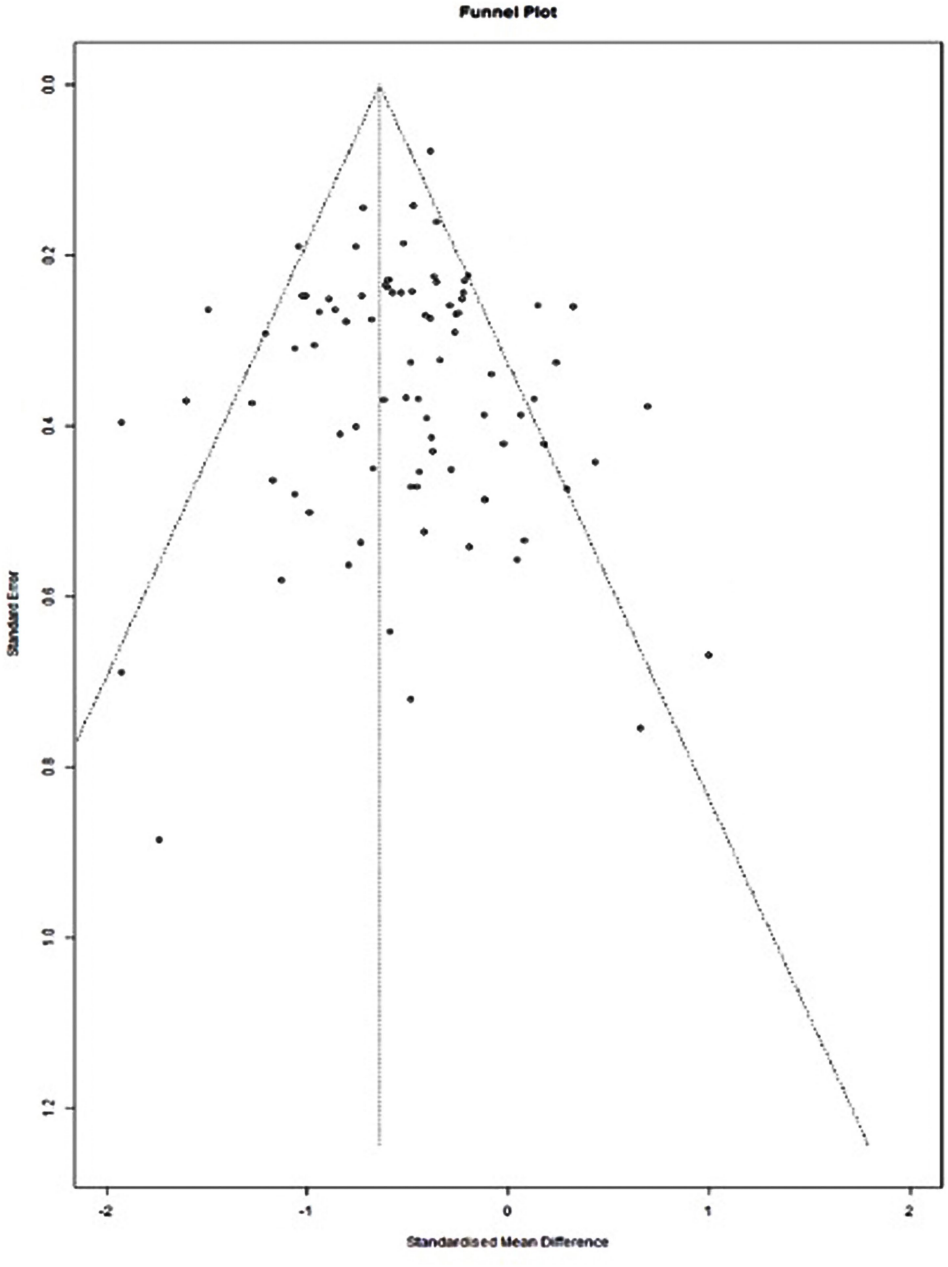
Funnel plot of the effects of distraction on reading comprehension in online environments.

Publication bias, the tendency for studies with statistically significant results to be preferentially published, poses a substantial threat to the validity of educational research meta-analytic conclusions. To assess potential bias, funnel plots were used to examine the symmetry of effect size distributions; asymmetry in these plots may indicate the omission of non-significant or small-sample studies from the published literature. Figure 3 displays a funnel plot of the effect sizes of reading comprehension, where the *y*-axis represents the standard error (indicating precision, with smaller values reflecting greater precision), and the *x*-axis represents the effect sizes. The vertical reference line indicates the pooled effect size, which reflects the expected distribution of studies in the absence of publication bias or small-study effects. Indicators of publication bias include asymmetry in the funnel plot, where effect sizes are unevenly distributed on either side of the pooled effect size, and a tendency for smaller, less precise studies (represented by higher SE at the bottom of the plot) to deviate more from the mean than larger, more precise studies. Although dissertations were included to reduce publication bias, the overall effect size remained negative. However, the distribution of effect sizes in the funnel plot is approximately symmetrical, with a concentration of high-precision studies (those with smaller SE) clustered near the top of the plot (Figure 3). This visual pattern indicates that the meta-analysis has no substantial publication bias because it reflects the expected distribution when both significant and non-significant results are adequately represented.

### 3.3 Main effects test

The R language was used to compute and analyze the data, including overall effect size, publication bias, subgroup differences, and other outcome measures, as presented in Table 3.

**Table 3:** Main effects.

Number of experiments performed	Hedges’*g*	95%-CI	*t*	*p*-value	tau^2^	*I* ^2^	*Q*
124	−0.64	[−0.89; −0.40]	−5.16	<0.001	1.3097 [1.3720; 2.6909]	88.3% [86.6%; 89.8%]	1054.43

Hedges’*g* is the standardized difference between two independent studies. The *p*-value (<=0.05) indicates that students in the distraction group achieved significantly lower grades than students in the control group.

Based on data from 124 experiments across 32 studies, participants exposed to attentional interference demonstrated significantly lower reading comprehension scores than those who were not exposed to attentional interference. The aggregated effect size across studies was Hedges’ *g* = −0.64, with a 95% confidence interval (CI) of [−0.89, −0.40], indicating a moderate to strong negative effect. A two-sample *t*-test comparing reading comprehension scores between the experimental and control groups yielded a statistically significant result (*t* = −5.16, *p* < 0.001), confirming that the experimental group performed significantly worse than the control group. The *I*^2^ value for the included studies was calculated to be 88.3%, indicating that 88.3% of the total variability in effect sizes is attributable to differences between studies rather than random sampling error. This reflects a substantial heterogeneity level within the meta-analytic dataset. Given the significant heterogeneity observed across the included experiments, conducting subgroup analyses is justified to explore which moderating variables may have impacted reading comprehension outcomes and to help identify the sources of variability in the data.

### 3.4 Moderating the effects test

The following variables were identified as potential moderators for the moderating effects test: research design, type of attentional interference, level of conscious effort required, pacing (time control), text type, participant age, reading device, and publication date. Table 4 presents the meta-analysis results examining these moderators.

**Table 4:** Effects of distraction on reading comprehension in online environments.

Moderator	Category	*n*	Hedges’ *g*	95%-CI	*Q*	*p*-value
Research design		124	−0.64	[−0.89, −0.40]	5.91	0.015
	Between-group	89	−0.81	[−1.16, −0.46]		
	Within-group	35	−0.32	[−0.52, −0.11]		
Distraction type		124	−0.64	[−0.89, −0.40]	8.46	0.076
	TV	59	−0.82	[−1.28, −0.36]		
	Music	25	−0.82	[−1.54, −0.10]		
	Noise	19	−0.48	[−0.76, −0.21]		
	IM	13	−0.30	[−0.53, −0.07]		
	Interrupt	8	−0.21	[−0.49, 0.07]		
Conscious effort		124	−0.64	[−0.89, −0.40]	0.30	0.584
	No	89	−0.59	[−0.88, −0.31]		
	Yes	35	−0.75	[−1.26, −0.24]		
Pacing		124	−0.64	[−0.89, −0.40]	0.11	0.742
	Limit	82	−0.61	[−0.90, −0.32]		
	No limit	42	−0.70	[−1.16, −0.24]		
Text type		124	−0.64	[−0.89, −0.40]	0.46	0.499
	Expository	86	−0.59	[−0.83, −0.34]		
	Narrative	38	−0.81	[−1.44, −0.18]		
Age		124	−0.64	[−0.89, −0.40]	19.00	0.105
	Primary school	3	−0.26	[−1.46, 0.95]		
	Junior high school	4	−0.27	[−0.72, 0.18]		
	High school	11	−0.25	[−0.88, 0.38]		
	University	106	−0.72	[−1.01, −0.44]		
Reading_device		124	−0.64	[−0.89, −0.40]	0.22	0.643
	Paper	84	−0.61	[−0.86, −0.35]		
	Computer	40	−0.75	[−1.35, −0.16]		

#### 3.4.1 Research design

Individual experiments were categorized based on the research design, which was classified as either a between-group or within-group design. When the research design was examined as a moderating variable, the *p*-value for the between-group difference was 0.0150, indicating a statistically significant moderating effect on reading comprehension outcomes. For studies using the between-group design, Hedges’ *g* was −0.81 with a 95% CI of [−1.16, −0.46], while for studies using the within-group design, Hedges’ *g* was −0.32 with a 95% CI of [−0.52, −0.11]. These results indicate that participants exposed to attentional interference in between-group designs demonstrated significantly lower reading comprehension scores than those in within-group designs. When the distribution type was examined as a moderating variable, the *p*-value for the between-group difference was 0.076, which was not statistically significant because it exceeded the conventional threshold of 0.05. Previous research has noted that reading comprehension tends to be less efficient under multitasking conditions than when full attention is dedicated solely to the reading task (Clinton-Lisell, 2021). Compared with participants not exposed to attentional interference in online environments, those in the experimental group, distracted by stimuli such as background music or TV, were more likely to have their attention diverted, resulting in reduced reading comprehension performance.

#### 3.4.2 Interference type

All experiments were grouped based on the type of attentional interference, which was categorized as watching TV, listening to music, background noise, IM, and interruption. Subgroup analysis revealed that the most negative impact on reading comprehension was experienced by the participants when the interference involved watching TV and listening to music, both yielding a Hedges’ *g* of −0.82. Conversely, the least negative effect was observed for interruption, with a Hedges’ *g* value of −0.20. Although these results indicate a trend implying variation in reading performance based on the type of interference, the overall moderating effect was not statistically significant (*p* = 0.076), as it exceeded the conventional threshold of *p* < 0.05. This indicates that while the interference type may influence reading outcomes, the evidence was insufficient to confirm a significant moderating effect within the current dataset.

#### 3.4.3 Individual consciousness

In this study, the experiments were grouped based on whether attentional interference required conscious effort, defined as the extent to which the subjects actively engaged in a secondary task during reading. Subgroup analysis was used to assess the moderating effect of this variable on reading performance, yielding a *p*-value of 0.574. Individual consciousness did not statistically moderate the relationship between attentional interference and reading comprehension, as the p-value exceeded the conventional threshold of 0.05. The effect size for conditions requiring effortful attention was Hedges’ *g* = −0.75, while the effect size for conditions not requiring conscious effort was Hedges’ *g* = −0.59, indicating only a minor, statistically non-significant difference in their impact on reading comprehension.

#### 3.4.4 Time control

Individual studies were categorized into subgroups based on the presence or absence of time limits, specifically distinguishing between time-constrained and time-unconstrained conditions. The subgroup analysis evaluated the moderating effect of time constraints on reading performance and yielded a *p*-value of 0.742, which exceeded the conventional significance threshold of *α* = 0.05. This indicates that time constraints did not significantly affect the reading comprehension outcomes of the participants. The effect size for participants in time-constrained settings was Hedges’ *g* = −0.61, whereas for those in time-unconstrained settings it was Hedges’ *g* = −0.70, further supporting the conclusion that time constraints had no statistically significant moderating effect.

#### 3.4.5 Text type

In this study, individual studies were grouped based on the type of article read, categorized as either expository or narrative. The meta-analytic synthesis of the differences between these groups yielded a *p*-value of 0.499, indicating that the text type was not a significant moderator of reading performance. This non-significant result indicates that the effect of attentional interference on reading comprehension did not differ significantly between expository and narrative texts, supporting the assumption of homogeneity across text-type subgroups. The effect size for the expository text group was Hedges’ *g* = −0.58, whereas that for the narrative text group was Hedges’ *g* = −0.81.

#### 3.4.6 Age

Age was treated as a moderating variable and categorized into four educational level subgroups: elementary, secondary, university, and adult learners. The subgroup analysis yielded a *p*-value of 0.105, indicating that age was not a significant moderator of reading comprehension outcomes at the conventional threshold (*p* < 0.05). However, the descriptive analysis showed that participants below the university level exhibited a smaller negative effect size (Hedges’ *g* = −0.20), whereas university-level participants had a more pronounced negative effect size (Hedges’*g* = −0.72). This indicates that college students may be more susceptible to attentional interference during digital reading than younger learners, possibly due to higher cognitive demands or increased exposure to multitasking contexts.

#### 3.4.7 Reading device

Individual experiments were categorized according to the type of reading device used: paper-based versus computer-based reading. The subgroup analysis yielded a *p*-value of 0.643, indicating no statistically significant difference between the two groups. The effect size for paper-based reading was Hedges’ *g* = −0.61, whereas for computer-based reading it was Hedges’ *g* = −0.75. These findings indicate that the type of reading device did not significantly moderate the effect of attentional distraction on reading comprehension in online environments.

#### 3.4.8 Year of publication

The results of the meta-regression analysis revealed that publication year was not a significant predictor of effect size variability, explaining only 0.35% of the total variance. In contrast, between-study heterogeneity accounted for 95.11% of the variability, indicating that the primary source of variation was differences in study-level characteristics—rather than temporal factors. These findings imply that from 2000 to 2025, distractions in digital reading environments have consistently had a detrimental impact on reading comprehension.

## 4 Discussion

Previous studies on the effects of attentional interference on reading comprehension in online environments have produced inconsistent results regarding the direction and magnitude of this relationship. To address this gap, the present study conducted a systematic meta-analysis of domestic and international research published since 2000, aiming to quantify the overall impact of attentional distractions on digital reading comprehension and to identify moderating factors contributing to differences across studies. The meta-analysis identified several significant moderators that influence the relationship between attentional interference and reading comprehension in digital contexts. These findings enhance existing literature by highlighting key factors that can guide future experimental designs and inform educational strategies aimed at reducing the negative effects of digital distractions on reading performance, including the controlled manipulation of distraction intensity.

### 4.1 Harmful effects of attentional interference on outcomes of digital reading comprehension

The accelerated advancement of digital tools has significantly transformed reading behaviors, especially in educational contexts, in the current era of rapid technological development. Consequently, digital reading has become the dominant mode of information acquisition, reflecting a broader shift toward digital literacy in modern learning environments (Dan, 2021). As the prevalence of digital reading continues to grow (Khatri, 2020), findings from the present meta-analytic synthesis reveal that attentional interference has an overall negative effect on reading comprehension in networked environments. When individuals engage in reading within a networked environment, attentional interference, such as sending and receiving IM, listening to music, or watching videos, diverts cognitive focus from the reading task. This shift in attention often contributes to lower reading comprehension scores and reduced overall performance. Attentional interference creates distractions, which in turn elevate cognitive load, making it more difficult for readers to maintain concentration and process textual information effectively (Schurer et al., 2023).

### 4.2 Study design, interference type, and age significantly influence reading comprehension outcomes

When the research design was examined as a moderating variable, the Hedges’ g for all studies using the between-groups design was −0.81, with a 95% CI of [−1.16, −0.46]. These findings indicate that participants affected by attentional interference in between-group studies scored significantly lower, indicating that the study design has a substantial impact on reading comprehension outcomes. Participants exposed to attentional interference in such designs likely experienced increased cognitive load, which diverted attention away from reading tasks and impaired comprehension. Regarding the mode of interference, different types of distractions were found to significantly influence digital reading performance. The meta-analytic synthesis showed that TV (Hedges’ *g* = −0.82) and music (Hedges’ *g* = −0.82), as forms of auditory–visual interference, produced the most detrimental effects on reading comprehension among all analyzed interference types.

These near-identical effect estimates indicate that both types of attentional interference disrupt cognitive processing to a similar extent, underscoring their substantial impact on reading performance in educational settings. Previous research has demonstrated that reading in an online environment elevates cognitive load, particularly when attention must be diverted from reading comprehension tasks to other competing activities (Wang, 2022; Luke and Jensen, 2023). Attentional interference is consistently associated with a marked reduction in reading comprehension outcomes. Background music, for instance, has been shown to increase the difficulty of semantic integration and disrupt cognitive neural processing during reading, as evidenced by changes in event-related potentials (Du et al., 2020). Additionally, eye-tracking studies have recommended that complex auditory stimuli, such as music, further elevate cognitive load, thereby impairing reading efficiency (Hao and Conway, 2022; Andreou and Gkantaki, 2025). When participants in the experimental group engaged in reading within an online environment, the presence of auditory distractions, such as TV or music, caused a shift in attention away from the reading task, thereby negatively impacting reading comprehension. These distractions increased the cognitive demands of the participants, requiring them to exert greater effort to maintain focus on the reading material (Chevet et al., 2022b).

In the analysis of age distribution, college students (including adults) experienced a greater negative effect of attentional interference on reading comprehension in online environments, indicating that reading comprehension performance declined more significantly in this group. This outcome may stem from the fact that individuals at the college level and beyond typically engage in more cognitively demanding reading tasks, which place a greater burden on working memory and require more sustained attention than those assigned to elementary or secondary school students. Consequently, attentional distractions in the online environment are more likely to disrupt reading efficiency (Schurer et al., 2023; Ronconi et al., 2025). Additionally, college students are typically exposed to more complex forms of attentional distraction than elementary and middle school students, who generally encounter simpler distractions. Hence, college students require a higher level of cognitive control to manage these interferences, which can lead to increased mental strain and reduced efficiency in processing reading tasks. Consequently, such distractions may exert a more pronounced negative effect on college-level reading comprehension (Luke and Jensen, 2023; Santhosh et al., 2024).

### 4.3 Individual consciousness, time control, text type, reading device, and year of publication do not significantly influence reading comprehension outcomes

Analysis of potential moderators at the individual (awareness), task (time constraints and article type), and environmental (reading device and publication year) levels revealed no statistically significant moderating effects on the relationship between attentional interference and digital reading comprehension. Statistical tests indicated that none of these variables significantly influenced reading scores, as all calculated effect sizes across subgroups remained within non-significant ranges. These findings indicate that the identified moderating variables did not significantly influence reading comprehension outcomes in online environments characterized by attentional distraction.

#### 4.3.1 Individual consciousness

Possible reasons for the absence of a significant moderating role of conscious effort in the effect of attentional interference on reading comprehension in the online environment include the following: First, reading in online environments tends to be more fragmented (Liu and Gu, 2020), and readers may subjectively perceive shallow reading as requiring low effort, even though the actual cognitive load may be high. Second, readers have become accustomed to frequent interruptions in digital settings, which may impair their ability to accurately assess cognitive exertion. This misalignment between perceived and actual cognitive load makes it difficult to capture the moderating effect of conscious effort on reading comprehension performance.

#### 4.3.2 Time control

Possible reasons for the absence of a significant moderating effect of time in the impact of distractions on digital reading comprehension include individual differences in cognitive abilities. Previous studies have shown that individuals with higher working memory capacity are better equipped to manage the adverse effects of time pressure on reading comprehension (Ramírez-Peña et al., 2022). Additionally, the current online reading environment, which is often marked by shallow and fragmented reading habits, may reduce the influence of time constraints on deep processing. Consequently, time limitations may not significantly affect comprehension outcomes, which explains the lack of a meaningful difference between time-constrained and time-unconstrained conditions.

#### 4.3.3 Text type

The absence of a significant moderating effect of text type on the relationship between attentional interference and reading comprehension in online environments may be attributed to the likelihood that readers employ metacognitive strategies to manage distractions. These strategies may help sustain comprehension performance across different text types, such as narrative and expository texts, thereby reducing the potential moderating influence of the article type (Lan et al., 2025).

#### 4.3.4 Reading device

Possible reasons for the lack of a significant moderating effect of the reading device on the impact of attentional interference in networked environments include the convergence of reading experiences across formats. Modern digital devices increasingly incorporate readability-enhancing features, enabling reading outcomes comparable to those of traditional paper-based formats (Schwabe et al., 2022). Additionally, users’ growing familiarity and frequent engagement with digital reading may reduce previously observed disadvantages associated with screen-based reading, thereby minimizing performance differences between device types (Chen et al., 2014).

#### 4.3.5 Year of publication

The absence of a significant moderating effect of publication year on the relationship between attentional interference and reading comprehension in online environments may be attributed to the relatively stable nature of distraction types and their cognitive impacts over time. Despite technological advances, the manner in which these distractions affect comprehension has been little changed, leading to consistent negative effects across studies from different years (Ronconi et al., 2025). This persistence underscores the ongoing need to develop and implement strategies to mitigate distractions in digital learning environments, regardless of the study year.

Notably, the absence of significant effects for individual consciousness, time control, type of article, reading device, and year of publication may be attributed to the relatively small sample sizes associated with these variables. Consequently, the observed effects were not statistically significant, making it inconclusive to assert that these moderators have no practical influence on reading comprehension.

## 5 Limitation

Digital reading has emerged as a prominent area of study over the past two decades; however, the overall research span remains relatively short, and large-scale investigations into moderating variables are still lacking. When examining specific subgroups and text types, sample sizes are often small, and studies incorporating key moderators, such as age, text type, and reading device, are limited. These statistical constraints may affect the accuracy of test results and the validity of research conclusions. Therefore, future research should focus on designing specialized and targeted studies that systematically investigate moderating variables, such as conducting experiments across different age groups to assess how attentional interference in the digital environment influences reading comprehension, followed by large-scale studies to further validate these effects.

Second, the broad definition of the online reading environment adopted in this study encompasses both paper-based and screen-based reading, which may limit the applicability of the findings to fully digital contexts (Salmerón et al., 2025). Therefore, future research should include rigorous empirical studies that specifically examine the impact of attentional distractions during screen-based reading in fully digital environments. Additionally, other potentially influential factors, such as screen type, participants’ cultural background, gender differences, research quality, and the nature of comprehension tasks, may significantly affect how distractions influence reading comprehension in online settings. However, these variables could not be comprehensively explored due to the limited availability of primary studies and the defined scope of this analysis. Future research should prioritize investigating these factors, particularly cultural background, using well-structured experimental designs.

In addition, the funnel plot used in this study as a visual tool to assess publication bias has certain limitations, as it may either underestimate or overestimate the true extent of bias, thereby affecting the robustness of the conclusions. To enhance accuracy in future analyses, researchers should complement funnel plot assessments with additional statistical methods, such as Egger’s regression test, Begg’s rank correlation test, or the trim-and-fill method, alongside quantitative measures and modeling techniques, to provide a more comprehensive and rigorous evaluation of publication bias.

## 6 Educational recommendations

In practical teaching applications, educators can employ cognitive load management strategies to help students minimize attentional distractions in online environments. First, to create more effective digital learning environments, educators and researchers should reduce distractions such as advertisements and social media notifications and develop interactive platforms that promote focused and self-regulated learning (Ronconi et al., 2025). Additionally, teachers can employ technologies such as eye-tracking to monitor students’ distraction levels and implement personalized interventions that help learners adopt self-regulation strategies to improve comprehension (Dontre, 2021; McCarthy and Hinze, 2021; Lan et al., 2025). Eye-tracking tools can also provide real-time insights into attentional behavior, allowing educators to adapt instructional approaches and enhance personalized learning support (Chen et al., 2021). Teachers can provide timely online feedback based on the performance of students to help them enhance their self-monitoring ability.

## 7 Conclusion

The central finding of this meta-analysis is that distractions in digital reading environments significantly impair reading comprehension performance, a result consistent with previous research (Liu, 2022; Wang, 2022; Luke and Jensen, 2023; O'Toole, 2024; Sun et al., 2024). Compared with traditional reading environments, digital reading contexts expose readers to a variety of multimodal distractions, such as hyperlinks, which alter attentional control mechanisms and increase cognitive load. This study is the first to synthesize the effects of specific moderators across different distraction types and study designs, thus providing a more nuanced understanding of attentional interference in digital reading contexts. This study incorporated the research design into the framework of moderating factor analysis. Through comparison, studies using a between-group design had significantly lower reading comprehension scores than those using a within-group design. Second, previous studies often focused on children and adolescents. This study explicitly included adult readers in the framework of moderating factor analysis. The results indicated that the reading comprehension ability of older college students (including adults) also declined in the online environment. This study refined the types of interference into five specific scenarios: instant messaging, exposure to external materials, watching TV, playing background music, and environmental noise. The research showed that reading comprehension ability was particularly affected when listening to music or watching TV, revealing the role of different types of cognitive neural mechanisms, such as listening to music and watching TV.

## Data Availability

The raw data supporting the conclusions of this article will be made available by the authors, without undue reservation.
